# Supplementary figures and images for: Parasite-induced IFN-γ regulates host defense via CD115 and mTOR-dependent mechanism of tissue-resident macrophage death
Source: PLoS Pathog. 2024 Feb 20;20(2):e1011502. doi: 10.1371/journal.ppat.1011502 (PMC10906828; doi:10.1371/journal.ppat.1011502)

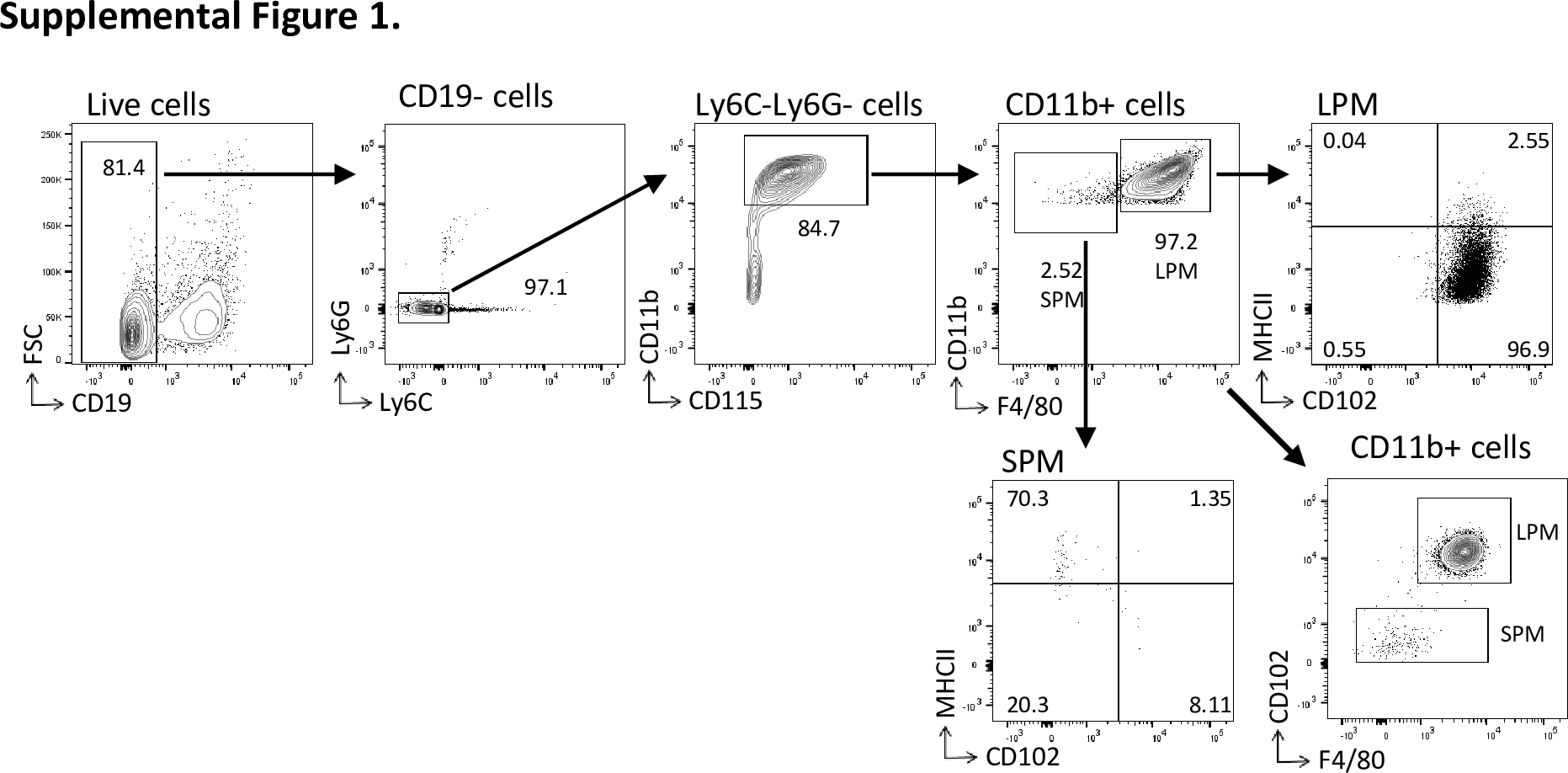

Supplement: S1 Fig — To identify peritoneal macrophage subpopulations, live (Zombie Yellow negative) single cells were gated for CD19- cells. LPMs were identified as F4/80+CD11b+ cells and SPMs were identified as F4/80-CD11b+ cells. LPMs and SPMs were additionally gated on CD102 and MHCII expression to validate the use of the alternate gating strategy for identifying LPMs as CD102+MHCII- cells and SPMs as CD102-MHCII+ cells. (TIF) [file ppat.1011502.s001.tif]

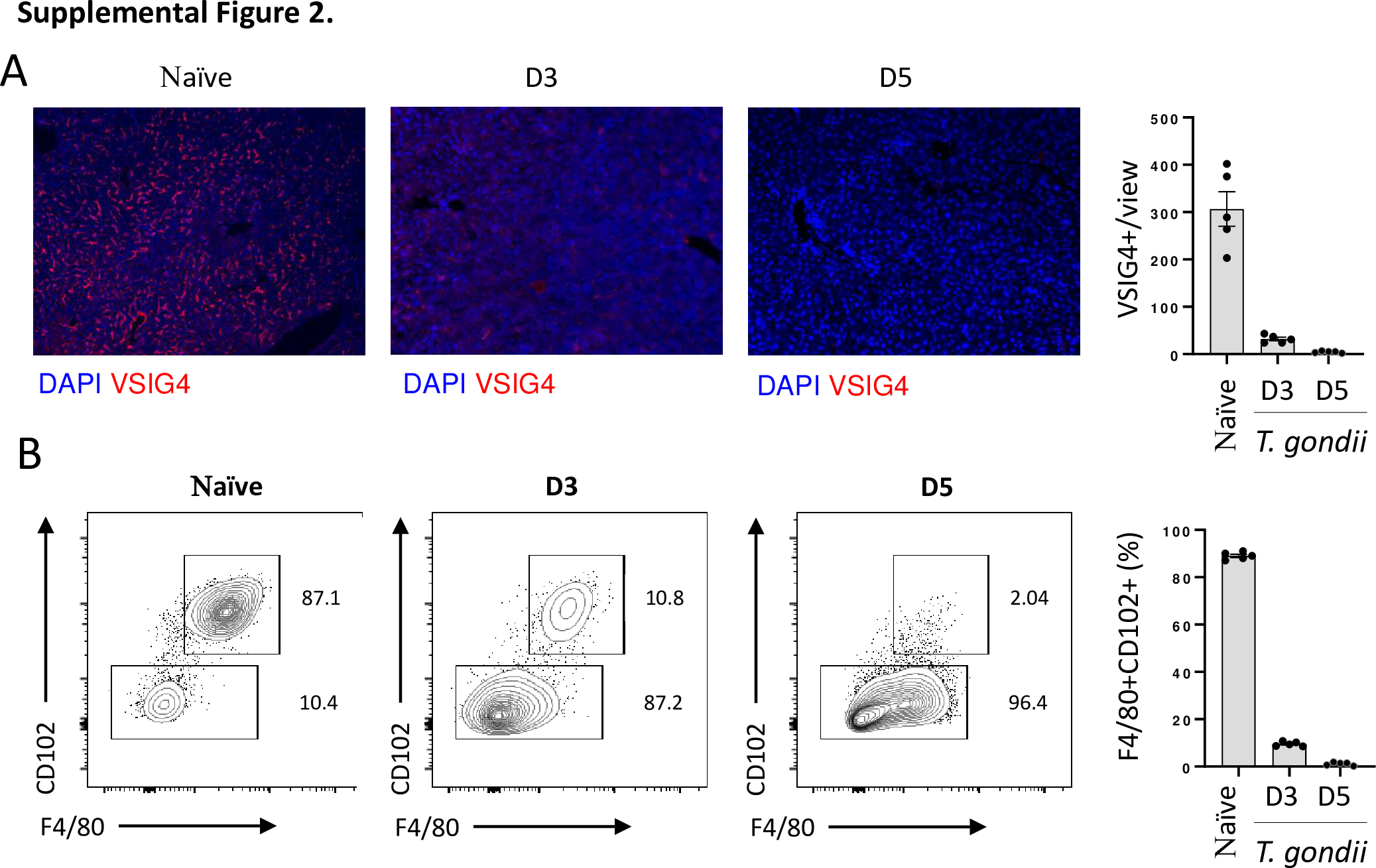

Supplement: S2 Fig — (A) Representative immunohistochemistry images and their quantification showing the expression of VSIG4 in liver on days 3 and 5 post intraperitoneal infection with 20 cysts of ME49 T. gondii. (B) Flow cytometric analysis of large (CD11b+F4/80+CD102+) and small (CD11b+F4/80-CD102-) peritoneal macrophages measured on days 3 and 5 post intraperitoneal infection with T. gondii. The results are representative of three independent experiments. (TIF) [file ppat.1011502.s002.tif]

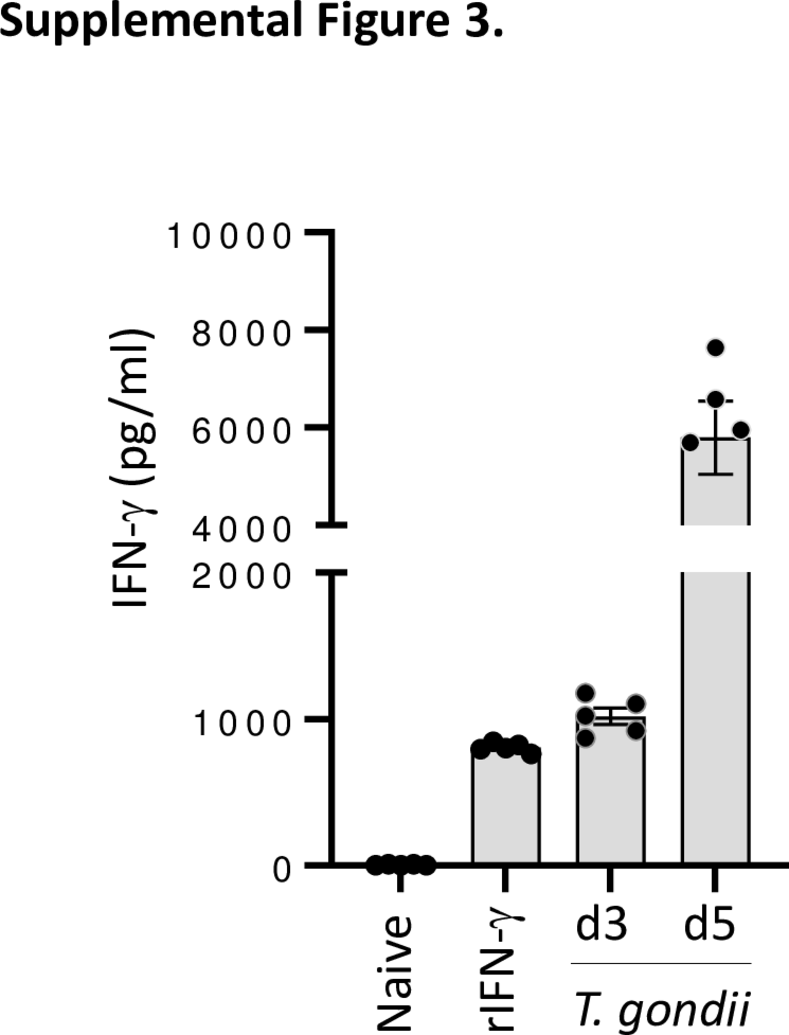

Supplement: S3 Fig — Mice were treated with recombinant 200 ng IFN-γ for 12 hours, and the peritoneal levels of the cytokine were analyzed by ELISA in comparison to T. gondii-infected mice (days 3 and 5 post intraperitoneal infection). The results are representative of three independent experiments. (TIF) [file ppat.1011502.s003.tif]

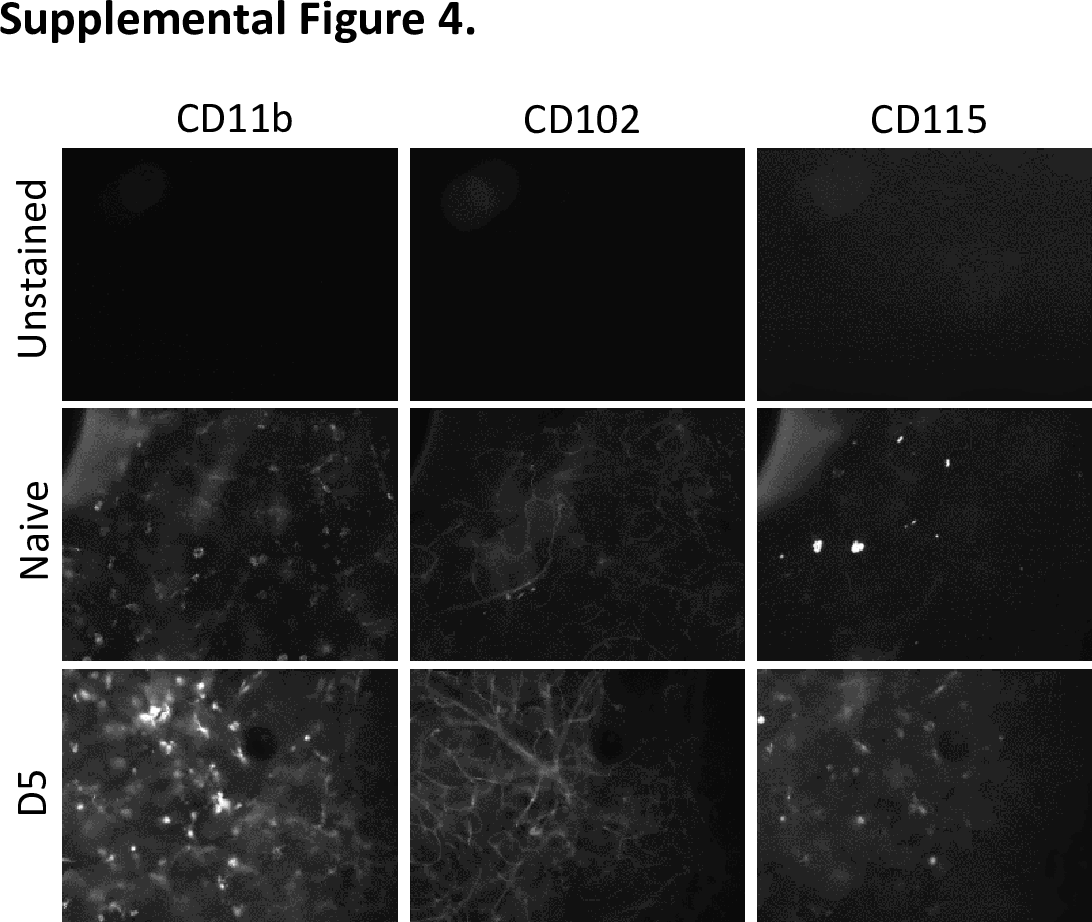

Supplement: S4 Fig — WT (C57BL/6) mice were either uninfected (Naïve) or infected intraperitoneally with 20 cysts of the ME49 strain of T. gondii for 5 days (D5). Entire omenta from the infected and controlled mice were analyzed by whole mount staining for the presence of CD11b, CD102, and CD115 expressing cells. The results are representative of three independent experiments. (TIF) [file ppat.1011502.s004.tif]

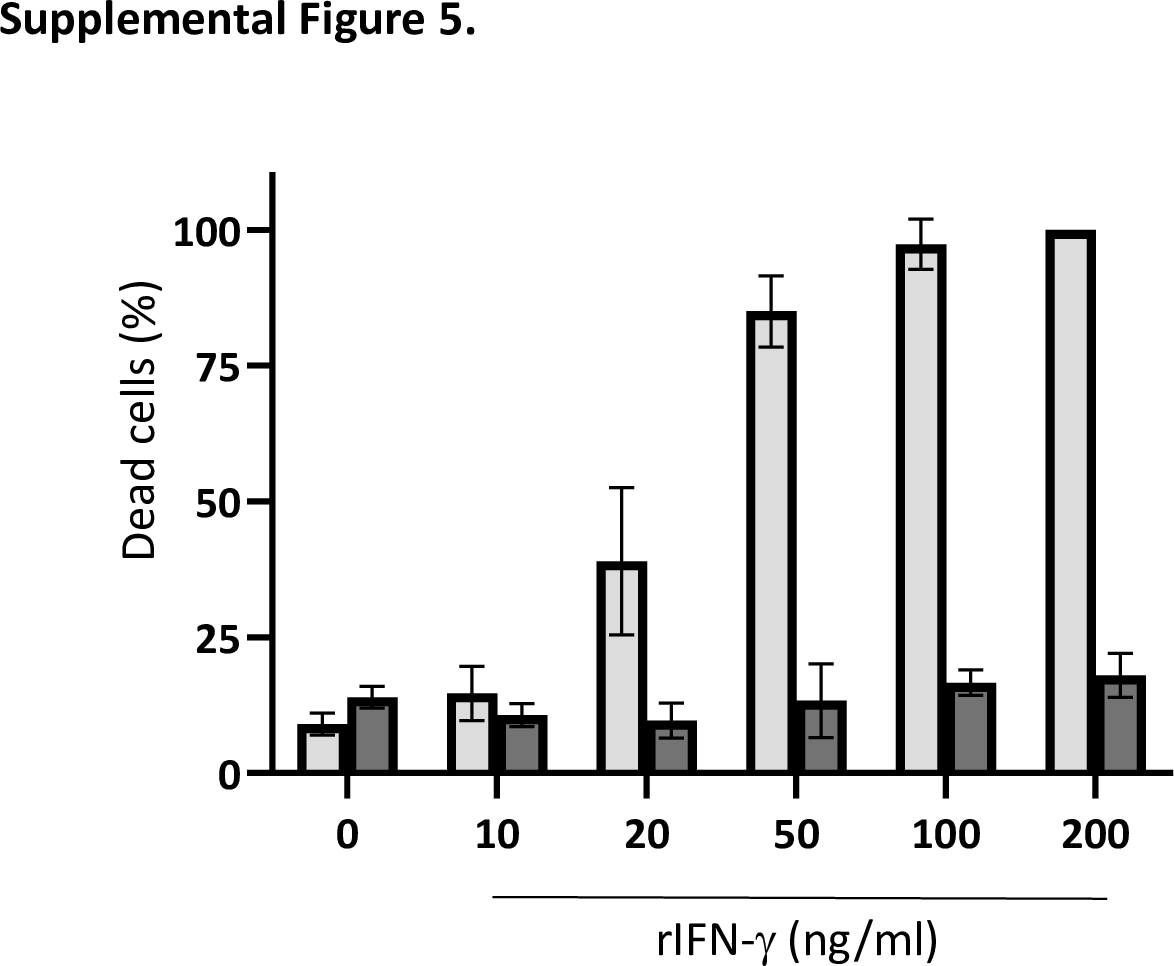

Supplement: S5 Fig — Cell-sort purified LPMs (CD45+ CD11b+F4/80+CD102+) and SPMs (CD45+ CD11b+F4/80-CD102-) were cultivated in a temperature, CO2, and humidity-controlled chamber in the presence of the indicated concentrations of rIFN-γ for 18 h. Dead or dying LPMs (grey bars) and SPMs (black bars) were detected by incorporation of Zombie Yellow. Error bars = mean ± SEM. The results are representative of three independent experiments. (TIF) [file ppat.1011502.s005.tif]

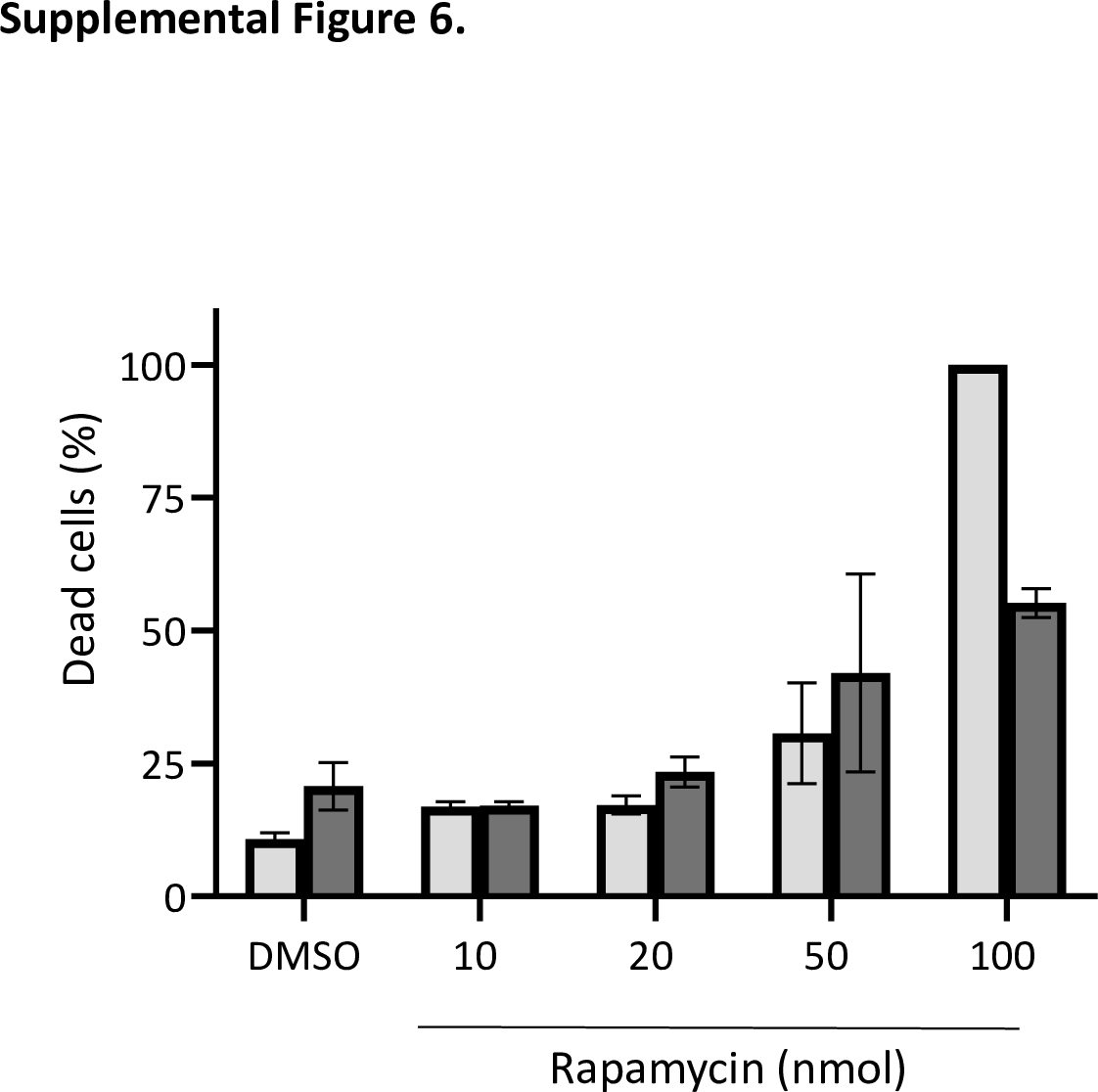

Supplement: S6 Fig — Cell-sort purified LPMs (CD45+ CD11b+F4/80+CD102+) and SPMs (CD45+ CD11b+F4/80-CD102-) were cultivated in a temperature, CO2, and humidity-controlled chamber in the presence of the indicated concentrations of rapamycin 18 h. Dead or dying LPMs (grey bars) and SPMs (black bars) were detected by incorporation of Zombie Yellow. Error bars = mean ± SEM. The results are representative of three independent experiments. (TIF) [file ppat.1011502.s006.tif]

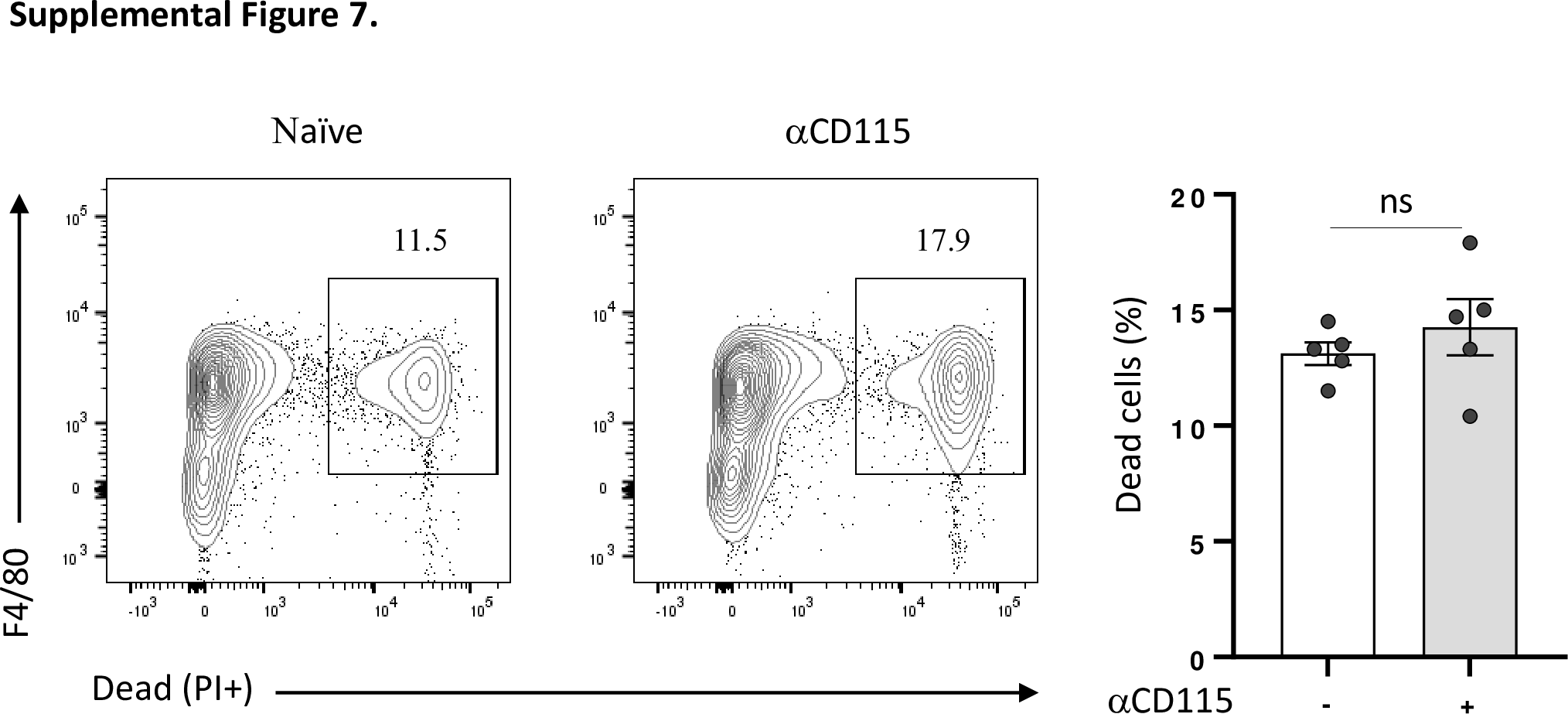

Supplement: S7 Fig — Purified peritoneal CD11b+ myeloid cells were cultured in a temperature, CO2, and humidity-controlled chamber in the presence of the αCD115 antibody for 18 hours. Dead or dying cells were detected by the incorporation of PI. Dead cells were quantified as PI+ cells. The results are representative of three independent experiments. (TIF) [file ppat.1011502.s007.tif]
